# Short-term exposure to ambient fine particulate matter and psoriasis: A time-series analysis in Beijing, China

**DOI:** 10.3389/fpubh.2022.1015197

**Published:** 2022-10-13

**Authors:** Junhui Wu, Hongbo Chen, Ruotong Yang, Huan Yu, Shaomei Shang, Yonghua Hu

**Affiliations:** ^1^School of Nursing, Peking University, Beijing, China; ^2^Department of Epidemiology and Biostatistics, School of Public Health, Peking University, Beijing, China; ^3^Medical Informatics Center, Peking University, Beijing, China

**Keywords:** fine particulate matter, outpatient visits, air pollution, psoriasis, time-series study

## Abstract

**Background:**

Ambient fine particulate matter (PM_2.5_) adversely affects human health and has been linked to a variety of skin disorders. However, little is known about the effects of PM_2.5_ on psoriasis.

**Methods:**

The Beijing Medical Claim Data for Employees database recorded 500,266 outpatient visits for psoriasis during 2010–2017. A generalized additive quasi-Poisson model was used to examine the relationship between daily PM_2.5_ concentrations and outpatient visits for psoriasis with stratification by sex, age, and season.

**Results:**

Short-term exposure to PM_2.5_ was associated with outpatient visits for psoriasis-related health concerns. A same-day increase of 10 μg/m^3^ in PM_2.5_ concentrations was associated with a 0.29% (95% confidence interval: 0.26–0.32%) increase in daily outpatient visits for psoriasis. Female and older patients appeared to be more sensitive to the effects of PM_2.5_ (*P* < 0.05).

**Conclusions:**

Short-term elevations in PM_2.5_ concentrations may be associated with exacerbations in psoriasis. Further work is warranted to confirm the findings and elucidate the underlying biological mechanisms.

## Background

Psoriasis is a common skin disorder and appears mainly in the form of chronic plaque psoriasis (psoriasis vulgaris) (1). Psoriasis is characterized by erythematous plaques covered by silvery lamellar scales, and common symptoms are pain, itching, and bleeding (2). Globally, psoriasis affects approximately 125 million individuals, and its prevalence is estimated at 3.2–8% (1, 3). The condition exerts significant physical, emotional, and social burdens on patients, including issues such as disfiguration, comorbidities (e.g., cardiovascular disease, psoriatic arthritis, and depression), and reduced ability to work (3, 4). There is no known cure for psoriasis (1), and the periodic recurrence and remission of symptoms consume substantial medical resources, especially in the outpatient department (1, 5). The underlying etiology of psoriasis is unclear, and may be a complex combination of immunological, environmental, and genetic factors that lead to clinically heterogeneous disease (6). Over the past few decades, an apparent upward trend in the prevalence of psoriasis has been observed in developed nations, coinciding with economic development and the accompanying environmental changes (7–10). Furthermore, several studies have reported that environmental factors such as airborne pollution increase the risk for skin disease (6). There is growing concern that air pollution may also have a detrimental effect on psoriasis.

Air pollution is an important and global risk factor for mortality, with an estimated 4.9 million deaths and 147 million years of healthy life lost annually (11). In recent years, multiple studies have reported significant associations between airborne pollution and skin disorders such as eczema, acne, and atopic dermatitis (12–14). However, the relationship between airborne pollution and psoriasis is uncertain. Among air pollutants, particulate matter with an aerodynamic diameter < 2.5 μm (PM_2.5_) has been identified as the greatest threat to human health (15–18), as the small particles can easily penetrate epithelial and endothelial cells, diffusing into blood and lymph circulation (19) and both inducing and exacerbating disease states (20, 21). Levels of PM_2.5_ pollution in China are among the highest worldwide and have been estimated to cause approximately 1 million premature deaths each year (22).

Some epidemiological studies have alluded to a possible link between PM_2.5_ and psoriasis, but not in detail. Previous studies conducted in Korea and Italy were conducted in low-level PM_2.5_ area, and their representation was limited (1, 2, 6, 23). In the present study, we hypothesized that short-term exposure to PM_2.5_ might contribute to the exacerbation of psoriasis. We used a time-series analysis to study the relationship between short-term exposure to PM_2.5_ and outpatient visits for psoriasis in Beijing, China.

## Methods

### Data collection

We collected daily PM_2.5_ concentrations from reports issued by the United States Embassy air monitoring station for the period between January 1, 2010 and December 31, 2017. These were the only consistent monitoring data we could access because China did not include PM_2.5_ in the air quality standard until 2013. Previous studies that compared PM_2.5_ levels recorded by this source and city-wide PM_2.5_ levels, found similar trends, and this data source has been used in multiple studies (24). The validity and reliability of the data have been demonstrated in detail in previous work (25) and are also described in Appendix S1. Daily mean temperature and relative humidity for the study period were obtained from the Chinese Meteorological Bureau. Personal identifiers and private information for the patients were removed for privacy; therefore, institutional review board approval and patient consent were not required.

Data on outpatient visits for psoriasis from January 1, 2010 to December 31, 2017 were obtained from the Beijing Medical Claim Data for Employees database, which covers all Beijing participants with basic medical insurance and includes all working or retired employees. The database records all medical claim data and basic demographics, date of visit, medications, and clinical diagnosis in Chinese and corresponding International Classification of Disease, 10th Revision codes. We used code L40.001 to identify cases of psoriasis. We only included patients with a primary diagnosis of psoriasis. Patients under 18 years were excluded. Beijing has a permanent population of ~21 million, of which more than 17.8 million (almost 85%) are included in the claim database. To control for potential residual confounding, we stratified the findings in subsequent analyses.

### Statistical analysis

We used a generalized additive quasi-Poisson model to evaluate the relationship between PM_2.5_ concentrations and psoriasis-associated outpatient visits. This model has been widely used and refined for air pollution and health-related time-series studies (26–29). Confounding covariates such as day of the week, public holiday, calendar time, temperature, and relative humidity were added to the main model (30, 31) and used as follows:


$$\begin{array}{lll} Log[E(Y_{t})] & = & \alpha +\beta PM_{2.5}+day\;of\;the\;week\\ & & +\;public\;holiday+s(calendar\;time,\;7\;per\;year)\\ & & +\;s(temperature,6)+s(relative\;humidity,3) \end{array}$$

where E(Y_t_) refers to the expected number of psoriasis-associated outpatient visits on day t, α represents the model intercept, β denotes the log (relative risk) of morbidity relative to unit increase in PM_2.5_ levels, and s() represents a smoothing based on the penalized splines. Holidays mainly include every weekend and three official traditional festivals. Following the approaches in several relevant studies (32, 33), we selected the degrees of freedom for calendar time, temperature, and relative humidity. To confirm the robustness of our findings, sensitivity analyses were conducted using various degrees of freedom (34).

We applied a penalized cubic regression spline for PM_2.5_ concentration, a widely used approach that can better achieve the purpose of evaluating the temporal correlation between PM_2.5_ concentrations and daily outpatient visits for psoriasis (35). Consistent with previous studies, and considering the skin directly exposed to PM_2.5_, we assessed the relationship between PM_2.5_ levels and psoriasis-associated outpatient visits by constructing models with a single-day lag from the current day (lag 0) up to the previous 3 days (lag 1, lag 2, and lag 3), and with 2-day (lag 0–1), 3-day (lag 0–2), and 4-day (lag 0–3) moving average concentrations. To examine effects in subgroups, we stratified the analyses by age (<65 and ≥65 years), sex, and season. The warm season was defined as April to September and the cool season was defined as October to March (36). Z tests were used to assess statistical differences between subgroups (37). The degrees of freedom and parameters set in the main model is consistent with those in subgroup analysis.

All findings are presented as the percentage change and 95% confidence interval in daily psoriasis-associated outpatient visits for each 10-μg/m^3^ increase in ambient PM_2.5_. We used the “mgcv” and “nlme” packages in R 3.2.2 to analyze the data. Percentage change was calculated as (relative risk – 1) × 100. Statistical significance was defined as two-sided *P* < 0.05.

## Results

Table 1 lists the characteristics of the psoriasis patients in our study. During the study period (January 1, 2010 to December 31, 2017), a total of 500,266 outpatient visits for psoriasis were identified in the health claims database. More than half (56.93%) of the patients were men; 15.96% of the patients were older than 65 years, and 56.03% of visits occurred in the cool season.

**Table 1:** Characteristics of outpatient visits for psoriasis between January 1, 2010 and December 31, 2017 in Beijing, China.

**Variable**	**All year**	**Cool season**	**Warm season**
Outpatient visits	500,266	280,302 (56.03)	219,964 (43.97)
**Sex (%)**			
Male (%)	284,815 (56.93)	159,588 (56.93)	125,228 (56.93)
Female (%)	216,299 (43.07)	121,484 (43.07)	94,816 (43.07)
**Age (year, %)**			
18–64 (%)	420,445 (84.04)	235,302 (83.95)	185,144 (84.17)
≥65 (%)	114,010 (15.96)	64,540 (16.05)	49,471 (15.83)

**Figure 1:**
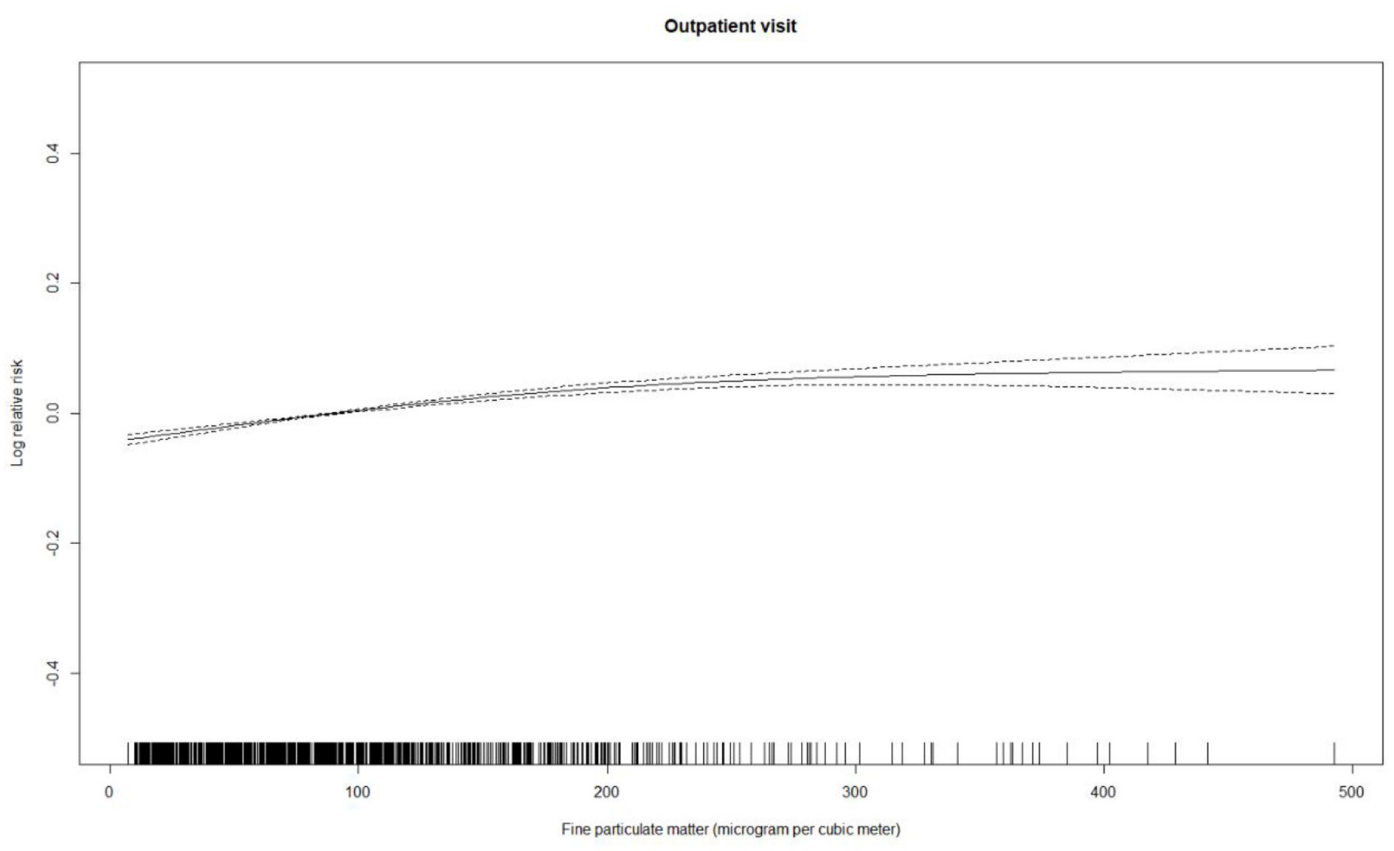
Concentration-response curve (solid line) for daily average concentrations of airborne fine particulate matter with three degrees of freedom and the log-transformed relative risk of outpatient visits for psoriasis between January 1, 2010 and December 31, 2017 in Beijing, China. The relative risk is adjusted for temperature, relative humidity, public holiday, and day of the week. Dotted lines represent the 95% confidence intervals.

Table 2 summarizes the descriptive statistics for the psoriasis-associated outpatient visits, PM_2.5_ concentrations, and weather conditions. The daily mean number of outpatient visits was 171 (standard deviation: 208). The annual average of PM_2.5_ concentrations was 86.8 μg/m^3^ (standard deviation: 74.3 μg/m^3^), with a maximum of 537.3 μg/m^3^. Figure 1 shows the association of PM_2.5_ concentrations with psoriasis-associated outpatient visits. There was a clear exposure-response association between the same-day (lag 0) daily average concentrations of PM_2.5_ and outpatient visits for psoriasis.

**Table 2:** Distribution of daily outpatient visits for psoriasis, fine particulate matter (PM_2.5_) concentrations, and meteorological conditions.

**Variable**	**Mean ±SD**	**Minimum**	**Percentile**			**Maximum**	**IQR**
			**25^th^**	**50^th^**	**75^th^**		
Daily outpatient visits	171 ± 208	7	58	100	256	1,413	198
PM_2.5_ (μg/m^3^)	86.8 ± 74.3	1.0	33.3	66.5	115.0	537.3	81.7
Daily outpatient visits during the cool season	165 ± 213	7	71	87	242	1,413	171
Daily outpatient visits during the warm season	180 ± 201	8	42	122	274	1,139	232
Temperature (°C)	14.6 ± 11.3	−14.3	2.6	15.1	24.0	34.5	21.4
Relative humidity (%)	51.8 ± 20.2	8.0	35.1	52.0	68.1	88.0	33.0

IQR, interquartile range; SD, standard deviation.

Table 3 presents the estimated relationships between acute elevations in PM_2.5_ concentrations and increases in outpatient visits for psoriasis. A 10-μg/m^3^ increase in PM_2.5_ levels corresponded to an increase of 0.29% (95% confidence interval: 0.26–0.32%) in same-day (lag 0) outpatient visits for psoriasis.

**Table 3:** Increases in outpatient visits for psoriasis associated with a 10-μg/m^3^ increase in levels of airborne fine particulate matter for various lags.

**Lag day**	**Percentage change**	**95% confidence interval**	***P***
Lag 0 day	0.29	0.26–0.32	<0.001
Lag 1 day	0.02	−0.01–0.05	0.428
Lag 2 day	0.11	0.09–0.14	<0.001
Lag 3 day	0.10	0.08–0.12	<0.001
Lag 0–1 days	0.20	0.17–0.23	<0.001
Lag 0–2 days	0.23	0.19–0.26	<0.001
Lag 0–3 days	0.24	0.21–0.28	<0.001

Table 4 lists the subgroup estimates. The association between PM_2.5_ concentrations and psoriasis-associated outpatient visits was greater in women (0.35%, 95% confidence interval: 0.30–0.39%) and in older patients (0.41%, 95% confidence interval: 0.34–0.48%). Sensitivity analyses confirmed these results (Table 5). The changes in degrees of freedom for calendar time (6–9), temperature (5–8), and relative humidity (3–6) did not substantially change the findings, indicating that the association was robust.

**Table 4:** Increases in outpatient visits for psoriasis associated with a 10-μg/m^3^ increase in airborne fine particulate matter stratified by sex, age group, and season.

**Subgroups**	**Percentage change**	**95% confidence interval**	***P***	***^a^P***
**Sex**				0.017
Male	0.24	0.20–0.28	<0.001	
Female	0.35	0.30–0.39	<0.001	
**Age (year)**				0.017
18–64	0.27	0.23–0.30	<0.001	
≥65	0.41	0.34–0.48	<0.001	
**Season**				0.051
Cool	0.26	0.16–0.36	0.035	
Warm	0.90	0.82–0.98	0.001	

a *P*-value was obtained by Z-test for the difference between the two risk estimates derived from subgroup analyses.

**Table 5:** Percentage change in same-day (lag 0) outpatient visits for psoriasis associated with a 10-μg/m^3^ increase in airborne concentrations of fine particulate matter using various degrees of freedom (*df*) for calendar time, temperature, and relative humidity.

**Variable**	***df***	**Percentage change**	**95% confidence interval**	***P-*** **value**
Calendar time	6	0.29	0.26–0.32	<0.001
	7^*^	0.24	0.21–0.27	<0.001
	8	0.24	0.20–0.27	<0.001
	9	0.17	0.14–0.20	<0.001
Temperature	5	0.29	0.26–0.32	<0.001
	6^*^	0.33	0.30–0.36	<0.001
	7	0.31	0.28–0.34	<0.001
	8	0.29	0.26–0.32	<0.001
Relative humidity	3^*^	0.29	0.26–0.32	<0.001
	4	0.29	0.26–0.32	<0.001
	5	0.29	0.26–0.32	<0.001
	6	0.29	0.26–0.32	<0.001

* The *df* value used in this study model.

## Discussion

In this citywide time-series analysis, we found significant and positive associations between PM_2.5_ levels and outpatient visits for psoriasis. This introduces fine particulate matter as a factor in psoriasis exacerbation. To our knowledge, this is the first large-scale citywide research in China to comprehensively assess the acute effect of PM_2.5_ on psoriasis-associated outpatient visits. Although previous correlation studies have explored the association in some low-level PM_2.5_ areas, the results of our study are meaningful for the developing countries with more serious air pollution problem. Our findings provide new evidence that may assist in improving targeted intervention strategies for psoriasis.

In developed and Western nations, regular outpatient visits often require an appointment and treatments may not be available on the same day or at local clinics. Furthermore, as previous studies have indicated, some psoriasis patients present with mild symptoms (1) and do not require immediate medical attention or hospitalization, therefore electing deferred outpatient services. Currently, China lacks a general practitioner-based referral system (38). Regular patient visits to the outpatient departments of hospitals do not require an appointment and are made on a first-come, first-served basis (39). In 2014, 95% of hospital visits in China were outpatient visits (39, 40). This is the main manner by which Chinese patients obtain medical advice, and the daily count of outpatient visits is a good indicator by which to evaluate the association of air pollution and psoriasis. We included a citywide outpatient visit dataset to ensure the representativeness and authenticity of our findings.

We found that increases in PM_2.5_ concentrations correlated with outpatient visits for psoriasis. A 10-μg/m^3^ increase in PM_2.5_ levels corresponded to a 0.29% increase in same-day outpatient visits. Although the risk is relatively weak, the public health burden is extensive in China because of its large population and considerable air pollution.

To date, only two studies have investigated the effect of PM_2.5_ on psoriasis. A citywide survey in Korea found that every 10 μg/m^3^ increase in PM_2.5_ resulted in patient visit increases of 2.71% (95% CI 0.76–4.71; *P* < 0.01) (41). Another observational study with both case-crossover and cross-sectional design conducted in Italy also suggested that exposure to mean PM_2.5_ over 15 μg/m^3^ in the 60 days before assessment were associated with a higher risk of psoriasis area and severity index 5 or greater point worsening (adjusted odds ratio, 1.25; 95% CI, 1.0–1.57) (42). Despite some differences in study design and method, the evidence from these two studies strongly supports our study. Multivariate negative binomial regression analysis was conduct in the study of Korea rather than Poisson model because there were over dispersion of data. In addition, the health effects are greater at low-level air pollution area generally. These may account for the larger effect size in Korea. There are a lot of differences between the Italian study and our approach. For the study location, the Italian study was based on hospital data, which could provide a more detailed observation of psoriasis pathological indicators and incorporate more accurate disease outcomes. In addition, based on cohort data, this study could record patients' repeated hospital admissions in detail, which also enabled both case-crossover and cross-sectional design to be carried out. Although there are some differences in method, this study is consistent with our conclusions regarding the association between PM_2.5_ and psoriasis. Further studies with larger cohorts and different settings are needed to corroborate our findings.

Some modifiers of the relationship between PM_2.5_ pollution and psoriasis-associated outpatient visits were considered. In our study, the adverse effects of PM_2.5_ were more pronounced in older patients. Similar findings have been reported for other skin diseases (43, 44) and may reflect age-related differences in inflammatory or immune responses. In addition, older individuals are more vulnerable to high levels of airborne particulate pollution (45). Furthermore, a decline in skin barrier functions associated with aging may lead to increased skin sensitivity to environmental irritants, pathogens, and allergens (46). In the present study, we observed higher estimates in female patients. Duvetorp et al. reported that women with psoriasis were more likely to experience anxiety and depression because of the symptoms, resulting in more frequent hospital visits when the disease worsened (47). From our findings, we recommend that older and female patients with psoriasis reduce their personal skin exposure during periods of severe PM_2.5_ pollution, even though we did not find significant differences in the effect of PM_2.5_ concentrations on psoriasis between seasons. The relationship between season and health effects should be investigated further.

Although our findings suggest that PM_2.5_ pollution is related to psoriasis exacerbations, little is known about the underlying biological mechanisms. Research indicates that multiple mechanisms may mediate the adverse effects of air pollution on skin by influencing skin microflora, activating aromatic hydrocarbon receptors, and inducing inflammatory responses and oxidative stress (48). These mechanisms may be similar in the pathogenesis of psoriasis (6). For example, there is evidence that the adverse effects of polycyclic aromatic hydrocarbons in PM_2.5_ are mediated by activation of the aryl hydrocarbon receptor and may lead to autoimmune conditions (48). Transcriptomic analysis has shown that airborne PM_2.5_ may affect the expression of psoriasis-related genes and pro-inflammatory cytokines and exerts adverse effects on human keratinocytes (49). Moreover, a study in human embryonic stem cells reported that PM_2.5_ can disrupt keratinocyte differentiation and the expression of genes related to inflammation and psoriasis (21). Overall, the mechanism by which PM_2.5_ affects psoriasis requires detailed study.

Our study has some limitations. First, it is an ecological study and individual exposure was not determined. Second, because the information was limited, we did not investigate other potential modifiers, such as comorbidities and nutrition, which may be associated with psoriasis. And different stratification by age may also cause bias, and more studies are needed to further explore the association in different age subgroups. Third, the PM_2.5_ monitoring data were provided only by one source, and the lack of authoritative records of other air pollutants limits our investigation of the independent effects of PM_2.5_, requiring verification of the findings. Fourth, because the lack of recent statistical data related to the incidence of psoriasis in Beijing city, and in our study a same person may have multiple visits, it is difficult to calculate the population attributable fraction accurately, more studies are needed to further explore these issues.

## Conclusion

The present study provides robust evidence that short-term elevations in PM_2.5_ levels are related to the risk of psoriasis. Long-term observations, estimates of personal exposure, and consideration of other pollutants and lifestyle factors are required.

## Data availability statement

The data analyzed in this study was obtained from private datasets. Requests to access these datasets should be directed to yhhu@bjmu.edu.cn.

## Author contributions

JW contributed to the study concept, had full access to all the data in the study, take responsibility for the integrity of the work as a whole, and from inception to published article. RY, HY, JW, and HC contributed to the statistical analysis and tables' development of this article. SS and YH interpreted the findings and drafted the article. All authors contributed to the critical revision of the article for important intellectual content.

## Funding

This work was supported by the National Natural Science Foundation of China (Nos. 81872695 and 81972158), National Key Research and Development Program of China (Nos. 2020YFC2008800 and 2020YFC2008801), and China Postdoctoral Science Foundation (No. 2022TQ0017). The funders were not involved in the research and preparation of the article, including study design; collection, analysis, and interpretation of data; writing of the article; no in the decision to submit it for publication.

## Conflict of interest

The authors declare that the research was conducted in the absence of any commercial or financial relationships that could be construed as a potential conflict of interest.

## Publisher's note

All claims expressed in this article are solely those of the authors and do not necessarily represent those of their affiliated organizations, or those of the publisher, the editors and the reviewers. Any product that may be evaluated in this article, or claim that may be made by its manufacturer, is not guaranteed or endorsed by the publisher.

## Abbreviations

CI: confidence interval
PM_2.5_: particulate matter with an aerodynamic diameter < 2.5 μm.
