# Supplementary material for: Short-term exposure to ambient fine particulate matter and psoriasis: A time-series analysis in Beijing, China
Source: Front Public Health. 2022 Oct 13;10:1015197. doi: 10.3389/fpubh.2022.1015197 (PMC9597881; doi:10.3389/fpubh.2022.1015197)
Supplement: Supplementary file 1 [file Table_1.DOCX]

**Supplementary Materials**

**PM_2.5_ and meteorological data**

Measurements of daily fine particulate matter (particulate matter with aerodynamic diameter ≤2.5 μm, PM_2.5_) concentrations during the study period were obtained from the United States (US) Embassy air-monitoring station, which was established on the rooftop of the embassy building located in Chaoyang district, Beijing. The air-monitoring station reported hourly PM_2.5_ concentrations using an automatic Met One BAM-1020 β attenuation monitor (Met One Instruments, Grants Pass, Oregon, USA) [1]. A previous study has indicated that the PM_2.5_ concentrations obtained from the station had approximately the same trend as citywide PM_2.5_ levels [2]. Until 2013, China has gradually introduced PM_2.5_ in the national air quality monitoring network and published real-time monitoring data. Therefore, data from the US embassy was the only publicly available source for daily PM_2.5_ measurements during the study period. To validate the exposure estimates using the US Embassy data, we checked the correlation between PM_2.5_ concentrations provided by the US Embassy and by official PM_2.5_ monitoring stations in Beijing for the whole year of 2014, and the result showed a R-square of 0.917 (Fig. S1). To reduce exposure misclassification, previous studies considered the maximum distance was approximately 40 km and have used the monitoring data as proxies for the personal exposure among individuals residing <40 km from the monitoring station [1, 3, 4]. Furthermore, 79.2% of Beijing’s total population and all areas with high population density (>5,000 people/km^2^) are within a 40-km radius of the air-monitoring station. The area also covers 97.8% (44/45) of the tertiary hospitals and 79.3% (69/87) of the secondary hospitals in Beijing [1]. The daily (24 hours) mean concentrations of PM_2.5_ were calculated and used as metrics for individual exposure levels. To control for the potential effects of weather conditions, meteorological variables including daily mean temperature (°C) and relative humidity (%) were collected from the Chinese Meteorological Bureau over the same period. Locations of the PM_2.5_ monitoring and meteorological stations are shown in Fig. S2.


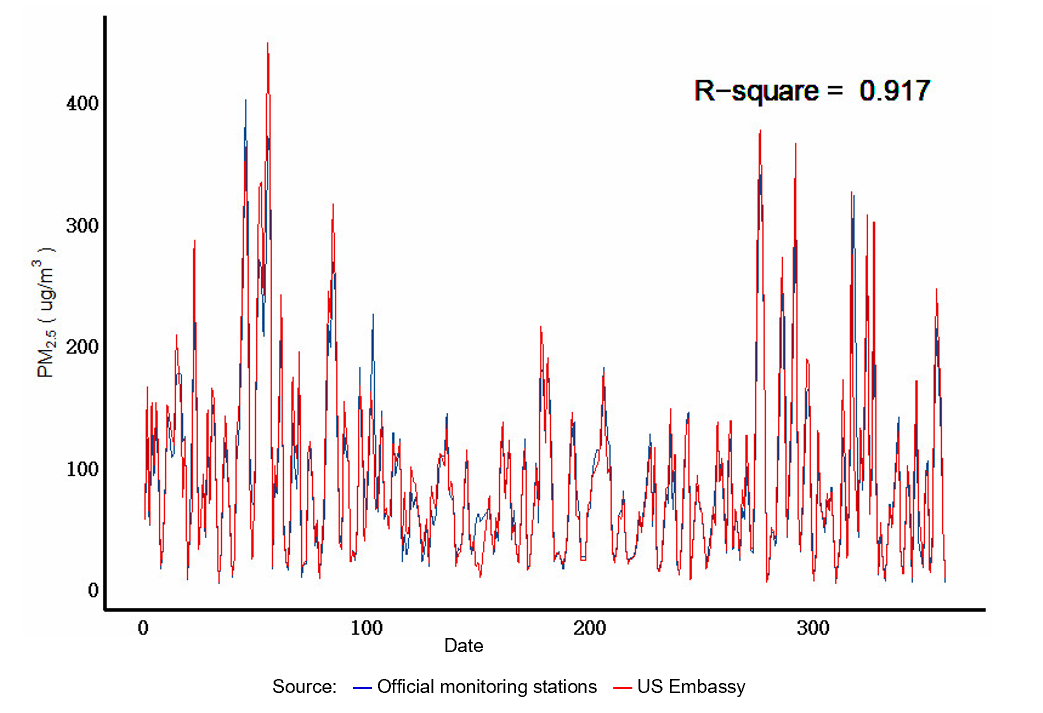


Fig. S1. The correlation between PM_2.5_ concentrations provided by the US Embassy and by the official monitoring stations in 2014.

Fig. S2. The locations of the PM_2.5_ monitoring station (in US Embassy) and meteorological station.

**References**

1. Xie W, Li G, Zhao D, Xie X, Wei Z, Wang W, Wang M, Li G, Liu W, Sun J *et al*: **Relationship between fine particulate air pollution and ischaemic heart disease morbidity and mortality**. *Heart* 2015, **101**(4):257-263.

2. Wang JF, Hu MG, Xu CD, Christakos G, Zhao Y: **Estimation of citywide air pollution in Beijing**. *PLoS One* 2013, **8**(1):e53400.

3. Dockery DW, Luttmann-Gibson H, Rich DQ, Link MS, Mittleman MA, Gold DR, Koutrakis P, Schwartz JD, Verrier RL: **Association of air pollution with increased incidence of ventricular tachyarrhythmias recorded by implanted cardioverter defibrillators**. *Environmental health perspectives* 2005, **113**(6):670-674.

4. Wellenius GA, Burger MR, Coull BA, Schwartz J, Suh HH, Koutrakis P, Schlaug G, Gold DR, Mittleman MA: **Ambient air pollution and the risk of acute ischemic stroke**. *Arch Intern Med* 2012, **172**(3):229-234.
